# Screening prognostic genes related to leucovorin, fluorouracil, and irinotecan treatment sensitivity by performing co-expression network analysis for colon cancer

**DOI:** 10.3389/fgene.2022.928356

**Published:** 2022-11-29

**Authors:** Pingping Wu, Xuan Pan, Kecen Lu, Ning Gu

**Affiliations:** ^1^ State Key Laboratory of Bioelectronics, Jiangsu Key Laboratory for Biomaterials and Devices, School of Biological Sciences and Medical Engineering, Southeast University, Nanjing, China; ^2^ Department of Medical Oncology, Jiangsu Cancer Hospital and Jiangsu Institute of Cancer Research, The Affiliated Cancer Hospital of Nanjing Medical University, Nanjing, China; ^3^ Jiangsu Cancer Hospital and Jiangsu Institute of Cancer Research, Nanjing Medical University, The Affiliated Cancer Hospital of Nanjing Medical University, Nanjing, China

**Keywords:** colon cancer, chemoresistance, WGCNA, prognosis hub genes, FOLFIRI

## Abstract

**Background:** Colon cancer is one of the most common malignant tumors in the world. FOLFIRI (leucovorin, fluorouracil, and irinotecan) is a common combination in chemotherapy regimens. However, insensitivity to FOLFIRI is an important factor in the effectiveness of the treatment for advanced colon cancer. Our study aimed to explore precise molecular targets associated with chemotherapy responses in colon cancer.

**Methods:** Gene expression profiles of 21 patients with advanced colorectal cancer who received chemotherapy based on FOLFIRI were obtained from the Gene Expression Omnibus (GEO) database. The gene co-expression network was constructed by the weighted gene co-expression network analysis (WGCNA) and functional gene modules were screened out. Clinical phenotypic correlation analysis was used to identify key gene modules. Gene Ontology and pathway enrichment analysis were used to screen enriched genes in key modules. Protein–protein interaction (PPI) analysis was used to screen out key node genes. Based on the Gene Expression Profiling Interactive Analysis (GEPIA) database, the correlation between the expression levels of these genes and the overall survival (OS) and disease-free survival (DFS) of colon cancer patients was investigated, and the hub genes were screened out. Immunohistochemistry of candidate hub genes was identified using the Human Protein Atlas database. Finally, clinical information and RNA sequencing data of colon cancer were obtained from The Cancer Genome Atlas project database (TCGA), the GEPIA, and the Human Atlas databases for validation.

**Results:** The WGCNA revealed that three hub genes were closely related to chemotherapy insensitivity of colon cancer: AEBP1, BGN, and TAGLN. The protein expression levels of AEBP1, BGN, and TAGLN in tumor tissues were higher than those in normal tissues. In addition, the gene expression levels of AEBP1, BGN, and TAGLN were negatively correlated with OS and DFS in colon cancer patients. Therefore, AEBP1, BGN, and TAGLN have been identified as potential biomarkers related to the response to FOLFIRI treatment of colon cancer.

**Conclusion:** We found that AEBP1, BGN, and TAGLN, as potential predictive biomarkers, may play an important role in the response to FOLFIRI treatment of colon cancer and as a precise molecular target associated with chemotherapy response in colon cancer.

## Introduction

As one of the most common malignancies, colon cancer is annually estimated to have 945,000 new cases all over the world (Weitz et al., 2005). With the development of tumor molecular biology and the application of new chemotherapeutics and molecular targeted drugs (Chasov et al., 2020), the treatment of colon cancer has made significant progress. However, the high rate of recurrence and metastasis impedes the prognosis of patients with advanced colon cancer. So, chemotherapy is indispensable in the clinical intervention strategy. These years have shown that fluorouracil (FU)-based chemotherapy serves as the standard treatment for patients with metastatic colon cancer. Nowadays, the NCCN (National Comprehensive Cancer Network) guidelines recommend the FOLFOX regimen as one of the standard adjuvant chemotherapy schemes for colon cancer. Irinotecan, as a crucial chemotherapeutic drug for the treatment of colorectal cancer, has been widely used in clinical practice and has a significant effect on the treatment of advanced colorectal cancer. The effectiveness of this single drug and the combination of fluorouracil and/or targeted drugs has been fully demonstrated. For patients with advanced colon cancer, the efficacy of the combination chemotherapy of FOLFOX (oxaliplatin, FU, and calcium folinate) and FOLFIRI (leucovorin, FU, and irinotecan) has been affirmed. The combination of FOLFIRI (leucovorin, FU, and irinotecan) is common in chemotherapy regimens. However, statistics show that the inactive rate of FOLFIRI is approximately 50% (Goldberg et al., 2004). The molecular biological mechanisms of insensitivity to FOLFIRI treatment are still to be investigated.

In chemotherapy sensitivity of colorectal carcinoma, inheritance is an essential factor. Mariadason et al. (2003) reported that the measurement of multiple marker genes performed a more accurate assessment of the response to chemotherapy. In addition, a study revealed that gene expression profiling might improve the response prediction efficiency of preoperative chemoradiotherapy in rectal adenocarcinoma (Ghadimi et al., 2005).

The application of the weighted gene co-expression network analysis (WGCNA) is popular, in terms of the construction of free-scale gene co-expression networks and analysis of large-scale gene expression profile datasets, which contributes a lot to identify modules of highly correlated genes (Langfelder and Horvath, 2008). The WGCNA has been successfully used to explore the relationship between gene sets and clinical tumor characteristics, which provides new approaches for tumor biomarker screening (Tian et al., 2020; Zhou et al., 2021).

In this paper, we studied the correlation patterns between genes through the WGCNA-based system biology methods and screened and finally determined new biomarkers related to the response to FOLFIRI treatment of colon cancer.

## Materials and methods

### Data procession

**FIGURE 1:**
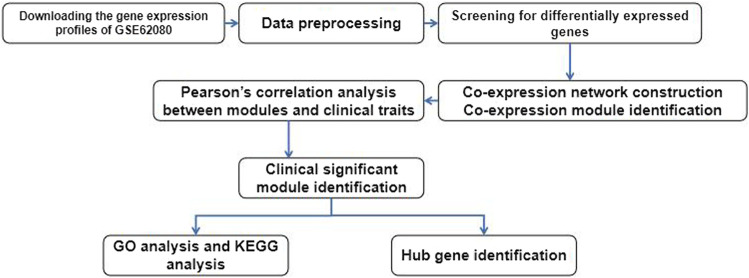
Flowchart of data preparation, processing, analysis, and validation.

Figure 1 reveals the process of our study. We downloaded the gene expression profiles of GSE62080, which were based on the GPL570 platform (Affymetrix Human Genome U133 Plus 2.0 Array) from the Gene Expression Omnibus (GEO) database (https://www.ncbi.nlm.nih.gov/geo/query/acc.cgi?acc=gse62080). We chose GSE62080 because this expression profile data include 21 samples from patients with advanced colorectal cancer, and all patients had received chemotherapy based on the FOLFIRI regimen. We used the robust multiple-array average (RMA) algorithm in the affy package of the Bioconductor in R to preprocess the gene expression profile data. We got the gene expression profile dataset containing 23,519 genes after background correction, quantile normalization, and probe aggregation. Then, 10,825 genes which were the top 50% most variant genes by analysis of variance were screened for further analysis.

### Co-expression network construction

After performing the WGCNA package in R, a gene co-expression network was constructed by the gene expression data profile from 10,825 genes screened before (Langfelder and Horvath, 2008). The following is the adjacency matrix aij to calculate the connection strength between each pair of nodes:

$$S_{i,j}=\left|cor\left(X_{i},X_{j}\right)\right|a_{ij}={S_{ij}}^{\beta }.$$

In this formula, vectors of the expression value for gene i and j were represented as X_i_ and X_j_. The Pearson correlation coefficient of gene i and gene j was shown as S_ij_. The power of β = 7 (scale-free R2 = 0.95) is adopted as the soft-thresholding parameter to ensure a scale-free network. For the hierarchical clustering of the weighting coefficient matrix, the genes with a high correlation were gathered in the same module and identified. We used the adjacency matrix to calculate the topological overlap measure (TOM), which represented the overlap in shared neighbors, and the functional modules of 10,825 genes were determined.

$${TOM}_{i,j}=\frac{\sum_{K=1}^{N}A_{i,j}\times A_{k,j}+A_{i,j}}{\min\left(K_{i},K_{j}\right)+1-A_{i,j}}.$$

In this formula, A is the weighted adjacency matrix given by ${A_{i,j}=\left|cor\left(X_{i},X_{j}\right)\right|}^{\beta }$ . β = 7 is the soft-thresholding parameter. Then, we set the minimum size of the gene group to be 100 for the gene dendrogram and conducted an average linkage hierarchical clustering based on the TOM-based dissimilarity measure. After that, the DynamicTreeCut algorithm was performed to classify genes with similar expression profiles into the same gene modules.

We used $1-{TOM}_{i,j}$ as the distance to perform hierarchical clustering of genes and then identified modules based on the dynamic pruning method. The most representative genes in each module are called module eigengene (ME), which represents the level of gene expression in the module. ME is the first principal component of each gene module. ME is calculated as follows:

$$ME=princomp\left({X_{i,j}}^{q}\right).$$

In this formula, q is the gene module. Then, we used the Pearson correlation between the expression profile of a certain gene in all samples and the expression profile of a certain ME to measure the module membership (MM) of this gene in the module. MM is calculated as follows:

$${{MM}_{i}}^{q}=cor\left(x_{i},{ME}^{q}\right).$$

Here, ${ME}^{q}$ represents the ME of the module q; ${{MM}_{i}}^{q}$ represents the MM of the gene i in module q. In addition, the gene significance (GS) was defined as the mediated *p*-value of each gene in the linear regression between gene expression and clinical characteristics. GS was used to measure the degree of association between genes and clinical characteristics. Moreover, we define the module significance (MS) as the average GS of all genes involved in the module.

### Gene Ontology and pathway enrichment analysis

The database g: Profiler (https://biit.cs.ut.ee/gprofiler/gost) was used for annotation, visualization, and integrated discovery. In this study, Gene Ontology (GO) and KEGG pathway analysis were technically supported based on the g: Profiler. GO analysis also included the biological process (BP), molecular function (MF), and cellular component (CC).The cut-off criterion of adjusted *p* < 0.001.

### Hub gene identification and validation

We measured the connectivity of genes by the absolute value of the Pearson correlation. Hub genes were defined as genes with high module connectivity (cor. gene module membership＞0.9). We measured the correlation between hub genes and certain clinical traits based on the absolute value of the Pearson correlation (cor. gene trait significance＞0.2). The clinical characters and RNA sequencing data were screened from The Cancer Genome Atlas project database (TCGA, https://cancergenome.nih.gov/). The edgeR package in R was used to normalize the mRNA sequencing data. Moreover, we used the Human Protein Atlas (HPA, http://www.proteinatlas.org) to identify associations of candidate hub genes and the immunohistochemistry of candidate hub genes. Survival analysis was exhibited on the basis of the Gene Expression Profiling Interactive Analysis (GEPIA) (Tang et al., 2017).

## Results

### Weighted co-expression network construction

**FIGURE 2:**
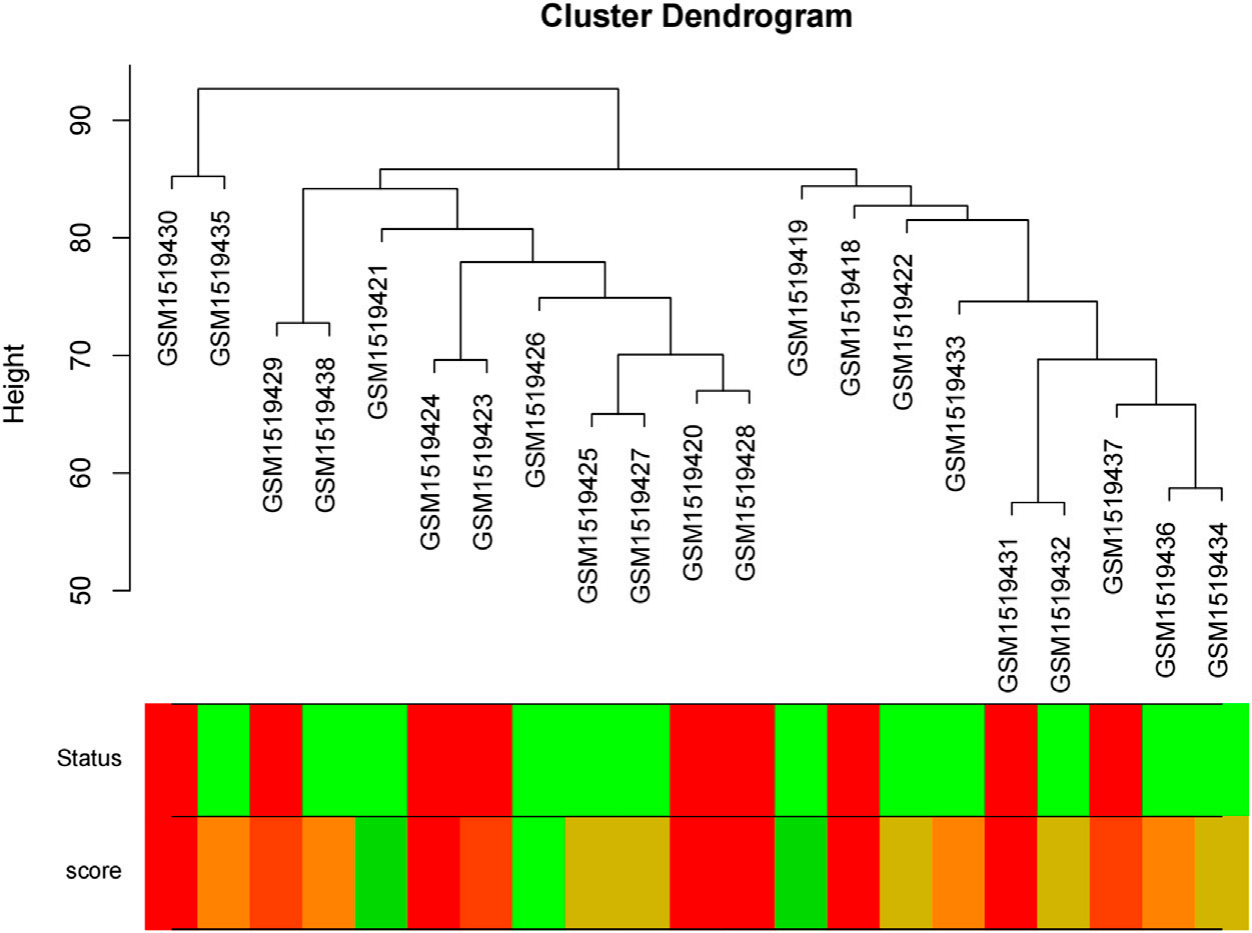
Clustering dendrogram of 21 samples.

**FIGURE 3:**
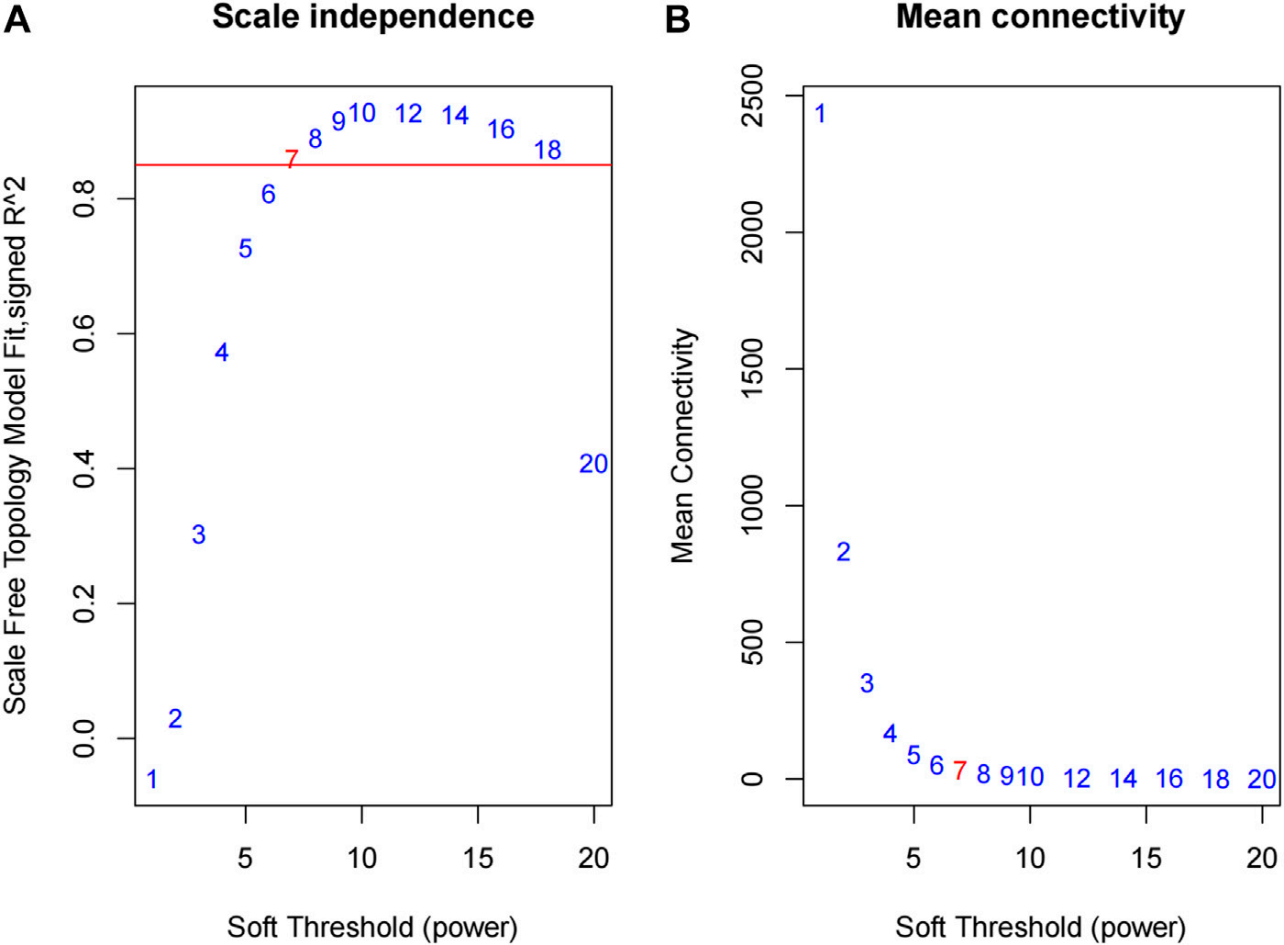
Determination of the soft-thresholding power in the WGCNA. **(A)** Analysis of the scale-free fit index for various soft-thresholding powers (β). **(B)** Analysis of the mean connectivity for various soft-thresholding powers.

**FIGURE 4:**
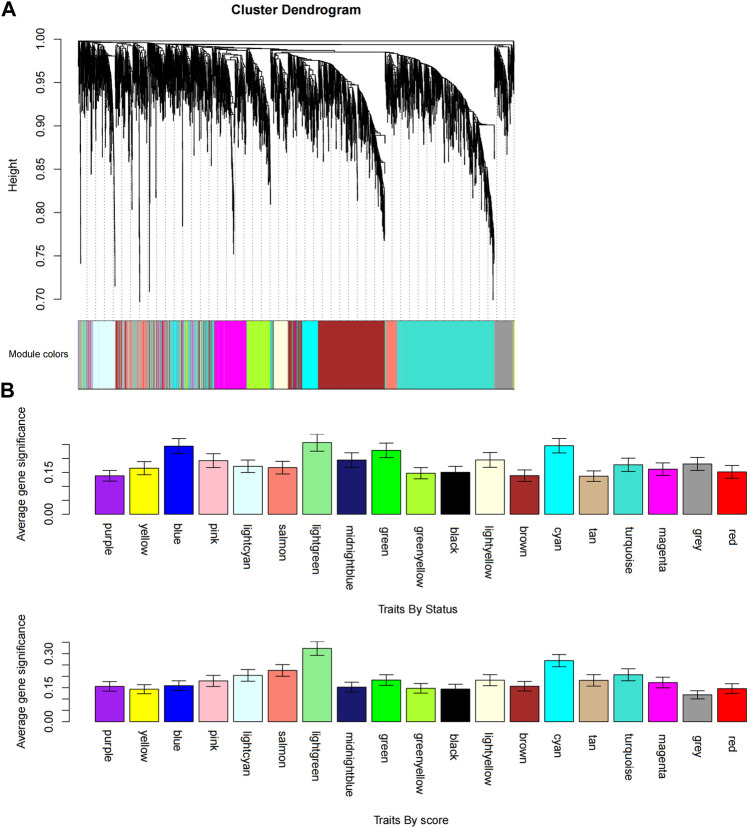
Identification of modules associated with the FOLFIRI response of colon cancer. **(A)** Dendrogram of all differentially expressed gene clusters based on a dissimilarity measure (1-TOM). **(B)** Distribution of the average gene significance and errors in the modules associated with status and scores of colon cancer.

We obtained the expression profile data which contained 21 samples and 21,648 genes. The variance of each gene in each sample was calculated, and the top 50% of genes with a small variance were eliminated. We analyzed and clustered the samples of GSE62080 based on the average linkage method and the Pearson correlation method. Figure 2 portrays the clustering results with sample characteristics. The status was divided into R (change in indicator lesion size＜−50%) and NR (change in indicator lesion size ≥ −50% or progression of disease). The scores were defined as follows: 5: change in indicator lesion size＜−75%; 4: −75% ≤ change in indicator lesion size＜−50%; 3: −50% ≤ change in indicator lesion size＜−25%; 2: −25% ≤ change in indicator lesion size＜0%; 1: change in indicator lesion size = 0%; and 0: progression of disease. We adopted the power of β = 7 as the soft-thresholding parameter to ensure a scale-free network (Figure 3). Then, we set the minimum size of the gene group to be 100 for the gene dendrogram and conducted the average linkage hierarchical clustering based on the TOM-based dissimilarity measure. The ME of each module was calculated, and 19 modules were identified (Figure 4).

### Clinical phenotypic correlation analysis and key module identification

**FIGURE 5:**
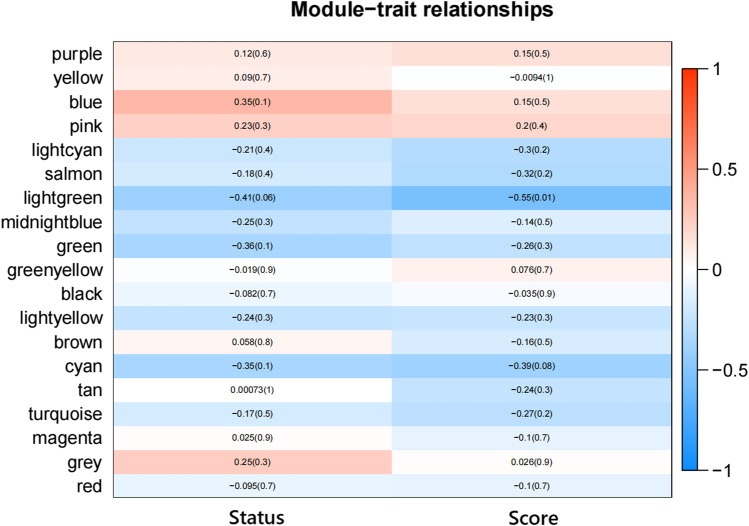
Heatmap of the correlation between module eigengenes and the FOLFIRI response of colon cancer.

**FIGURE 6:**
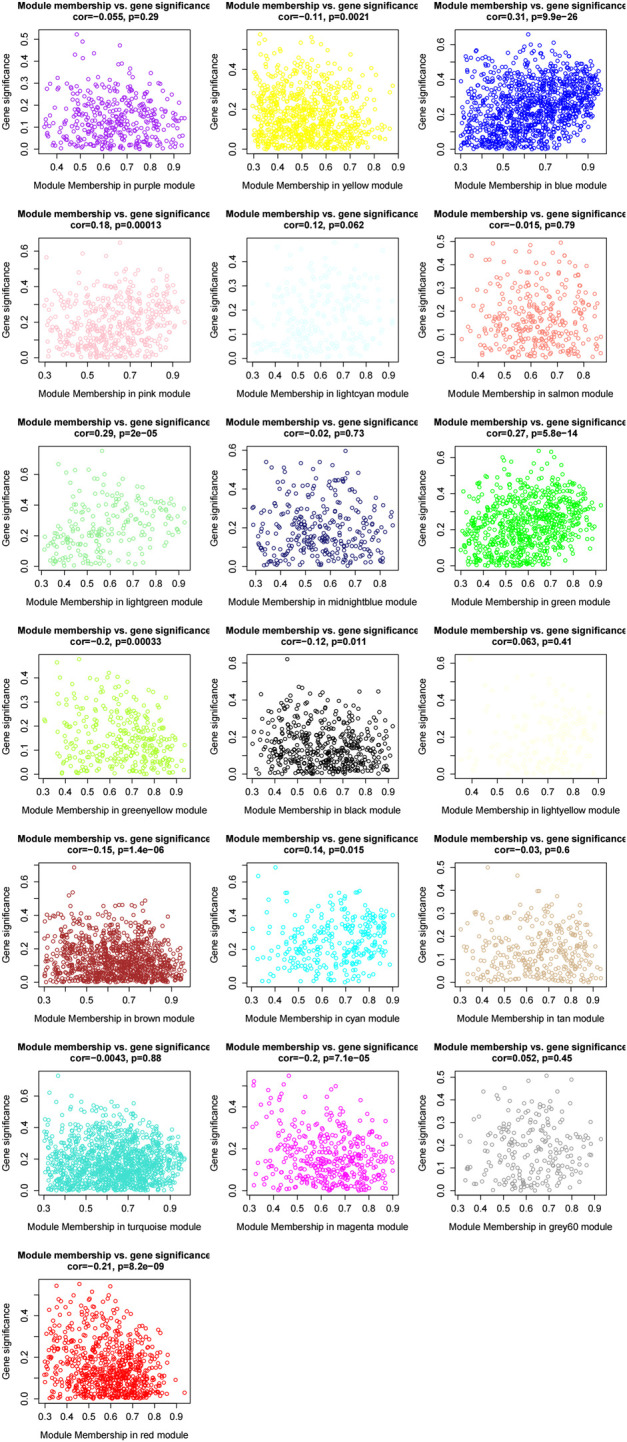
Distribution of gene significance in each module.

**FIGURE 7:**
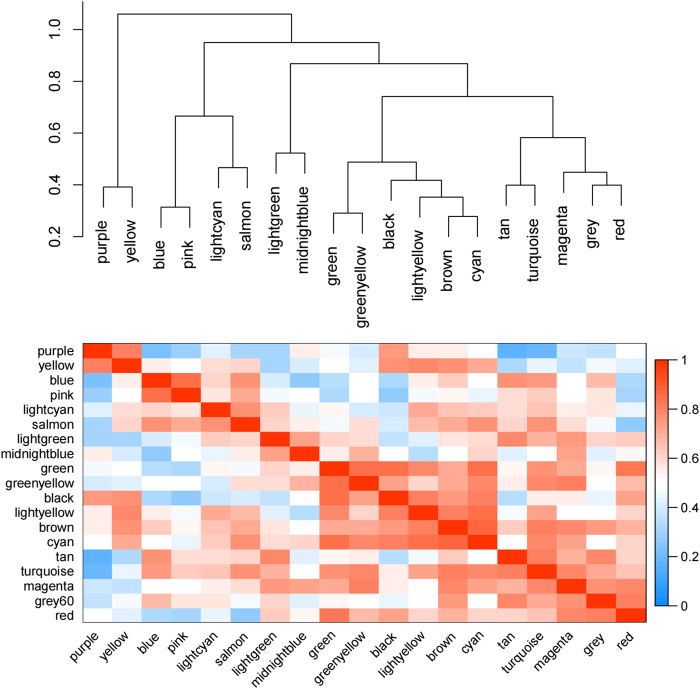
Correlation between 19 modules. The depth of red represents the strength of the correlation.

According to the eigenvectors of each module, we calculated the correlation between these modules and clinical phenotype and the result is shown in Figure 5. In the figure, the deeper the red color, the stronger the correlation, and the deeper the blue, the weaker the correlation. The figure revealed that the blue module had the strongest correlation with the significant response after FOLFIRI treatment. According to the expression level of each gene in each sample, we, respectively, calculated the correlation between the genes in these modules and status after FOLFIRI treatment. We separately counted the distribution of GS in each module (Figure 6). According to the ME of each module, we calculated the correlation between 19 modules (Figure 7). Through the aforementioned analysis results, the blue module was verified as having the highest correlation with the significant response after FOLFIRI treatment. Therefore, we take the blue module as a key module for further analysis.

### Gene Ontology and pathway enrichment analysis

**FIGURE 8:**
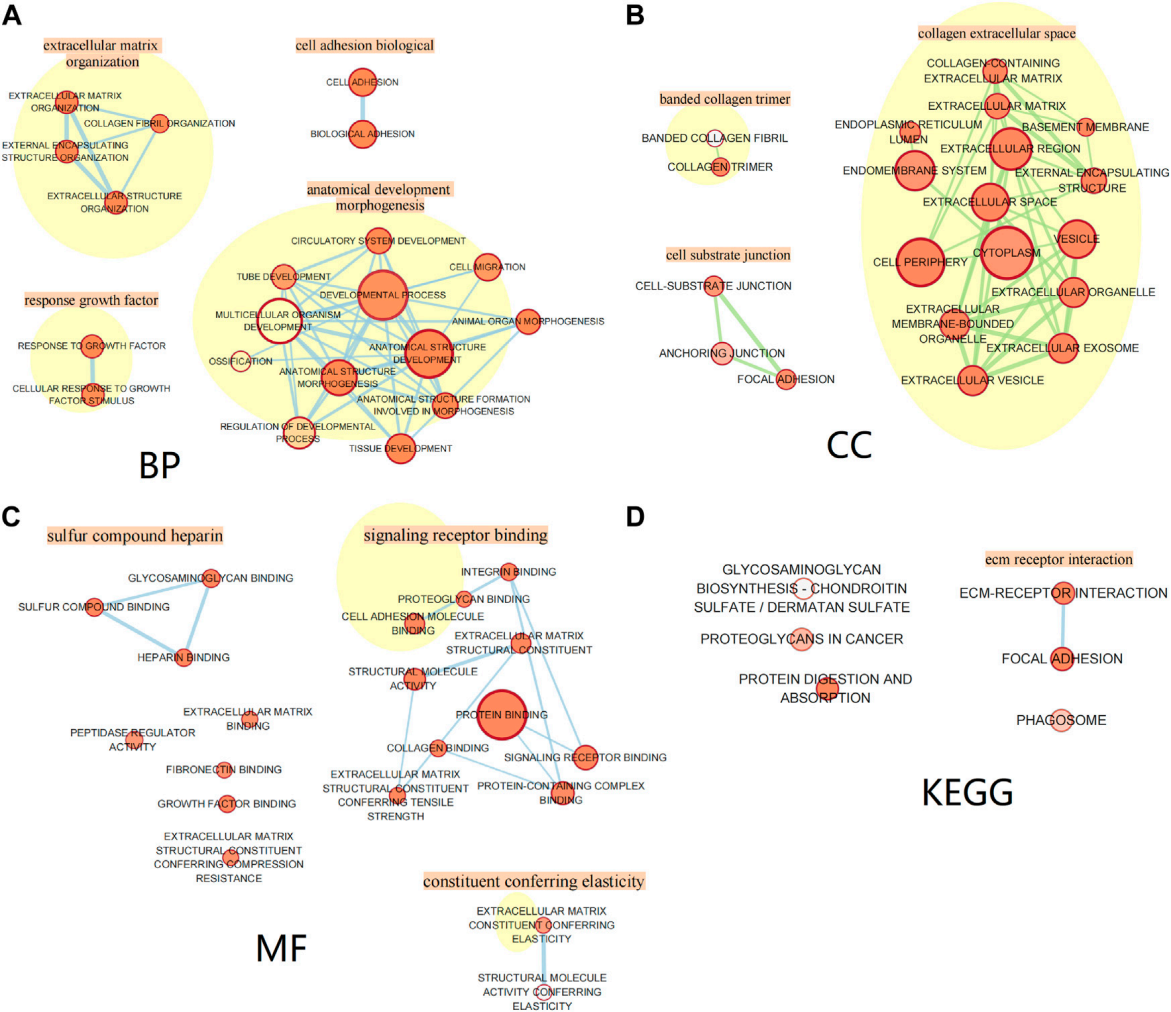
Gene Ontology and pathway enrichment analysis of the blue module genes. **(A)** Biological process analysis. **(B)** Cellular component analysis. **(C)** Molecular function analysis. **(D)** KEGG pathway analysis.

All genes in the blue module were divided into the biological process (BP) group, cellular component (CC) group, and molecular function (MF) group. We used Cytoscape (vision 3.8.2) analysis and screened out the top 20 functions of enriched genes in the BP, CC, and MF groups, respectively, and used the EnrichmentMap in Cytoscape for visualization of enrichment results. The genes in the BP group were mainly enriched in extracellular matrix organization, cell adhesion, growth factor response, and anatomical development morphogenesis. The genes in the CC group were mainly enriched in functions associated with collagen extracellular space, banded collagen trimer, and cell substrate junction. The genes in the MF group were mainly enriched in sulfur compound heparin, signaling receptor binding, and constituent conferring elasticity. Moreover, through Kyoto Encyclopedia of Genes and Genomes (KEGG) pathway analysis, we found that significant genes were mainly related to ECM–receptor interaction, glycosaminoglycan biosynthesis—chondroitin sulfate/dermatan sulfate, proteoglycans in cancer, protein digestion, and absorption. The results are shown in Figure 8.

### Identification and validation of hub genes

**FIGURE 9:**
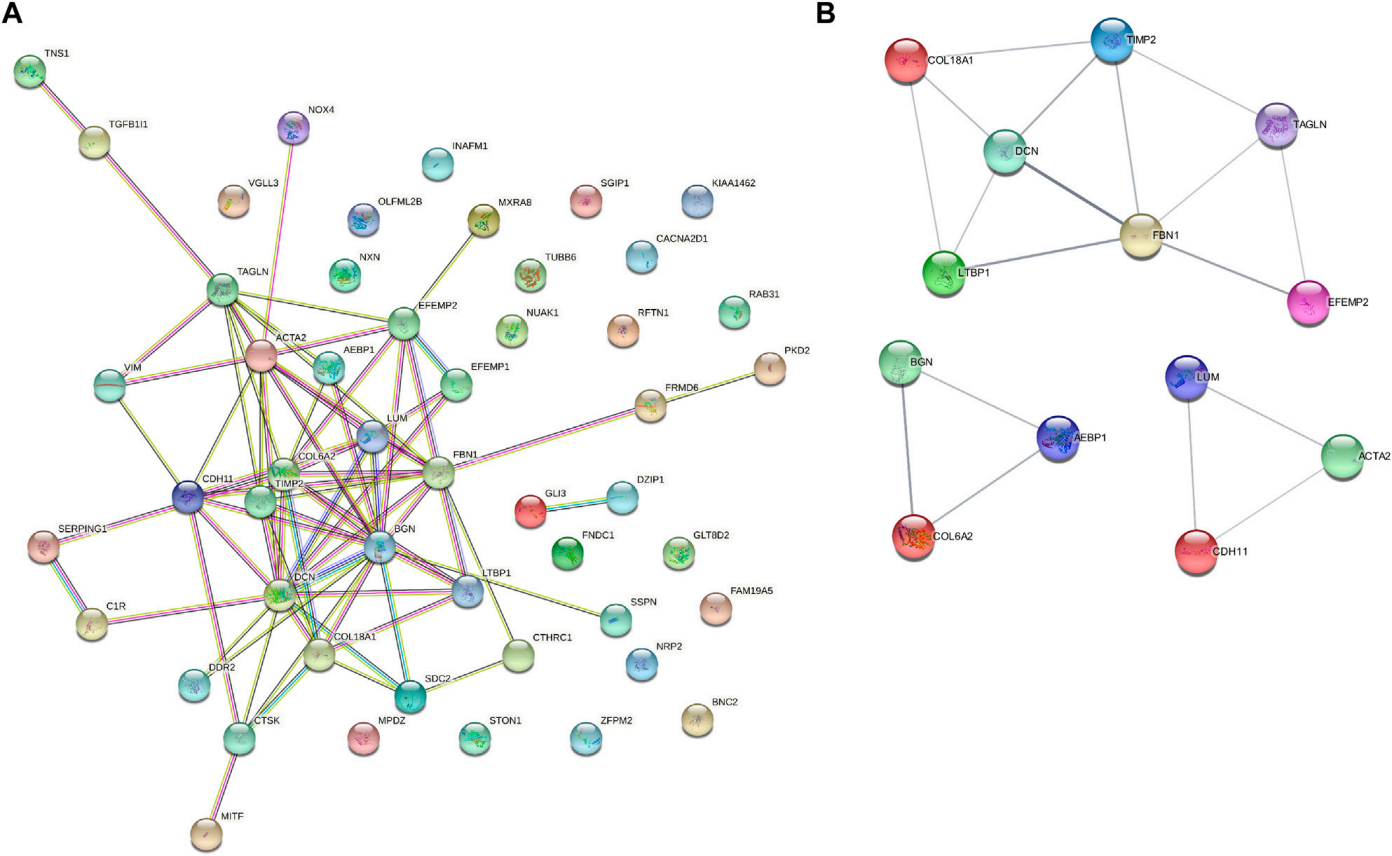
Protein–protein interaction (PPI) analysis of 50 hub genes and key node gene identification. **(A)** PPI analysis based on STRING. **(B)** Key node gene identification.

**FIGURE 10:**
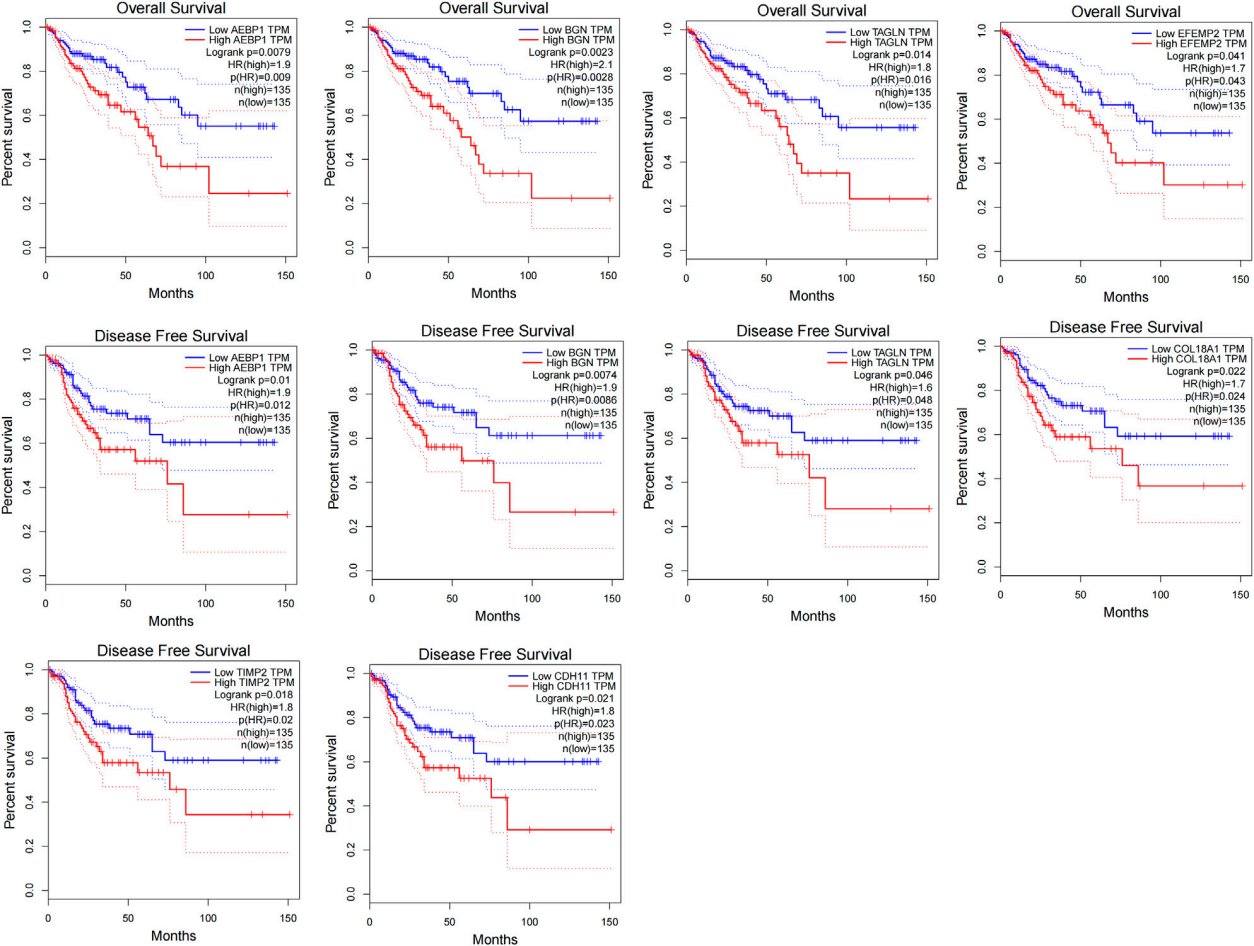
Disease-free survival analysis and overall survival of the 13 key node genes in colon cancer based on the GEPIA.

**FIGURE 11:**
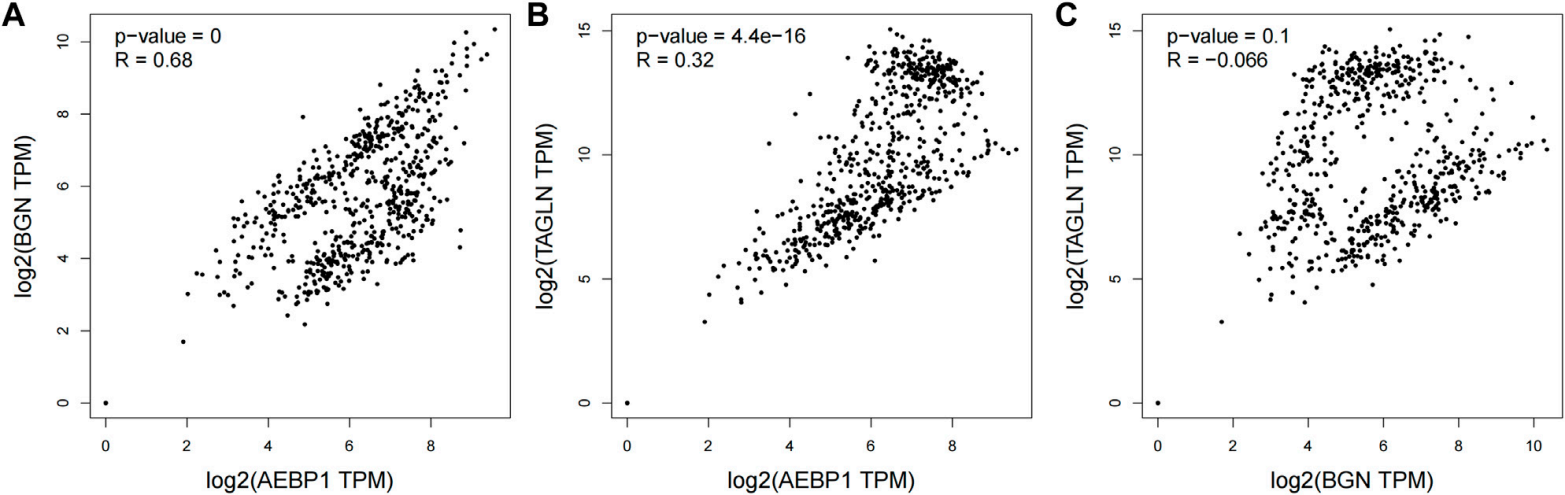
Correlations between AEBP1, BGN, and TAGLN.

**FIGURE 12:**
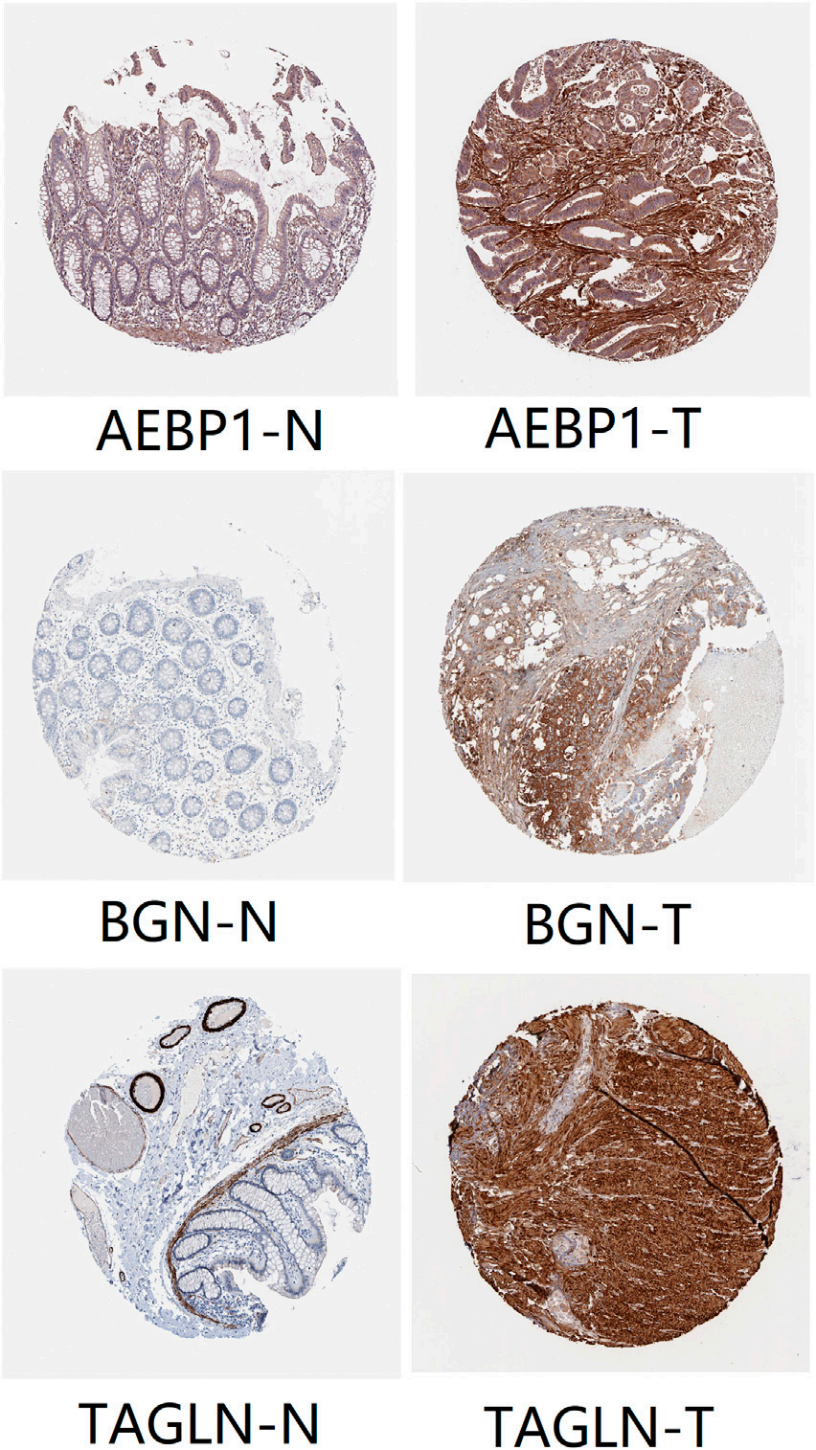
Protein expression levels of AEBP1, BGN, and TAGLN. “T” represents tumor, and “N” represents normal tissue.

We set the cut-off criteria as |MM| > 0.9 and |GS| > 0.2 and selected 50 hub genes with high connectivity in the blue module. Using STRING, we further performed protein–protein interaction (PPI) analysis on these 50 genes and then used MCODE in Cytoscope for further data screening and visualization. Finally, 13 key node genes (COL18A1, TIMP2, DCN, LTBP1, FBN1, TAGLN, EFEMP2, LUM, CDH11, ACTA2, BGN, COL6A2, and AEBP1) were screened out (Figure 9). We further explored the association of the expression levels of the aforementioned genes on overall survival (OS) and disease-free survival (DFS) in colon cancer patients in the GEPIA database. The results showed that the high levels of AEBP1, BGN, EFEMP2, and TAGLN expressions were associated with a shorter OS, and over-expression of AEBP1, BGN, CDH11, COL18A1, LUM, TAGLN, and TIMP2 were negatively associated with DFS (Figure 10). We selected three genes (AEBP1, BGN, and TAGLN) that were negatively related to both OS and DFS for further exploration. A significant correlation between AEBP1 and BGN expressions was found. In addition, AEBP1 and TAGLN expressions also showed a significant correlation. However, the correlation between BGN and TAGLN expressions was not statistically significant (Figure 11). According to the Human Protein Atlas database, we found that the protein expression levels of AEBP1, BGN, and TAGLN in tumor tissues were higher than those in normal tissues (Figure 12).

## Discussion

Colon cancer, as a common malignant tumor, seriously endangers people’s health. With the emergence of drug resistance after chemotherapy, improving the treatment response rate of advanced colon cancer has always been a key issue for clinical oncologists. This combination of irinotecan, fluorouracil, and leucovorin is reported as one of the first-line chemotherapies for advanced colon cancer with objective response rates of 49% and 56% (Douillard et al., 2000; Tournigand et al., 2004). Although studies have reported the existence of markers for predicting chemotherapy response, the clinical applications are still not routine. Thus, we need to explore precise molecular targets related to colon cancer chemotherapy response. In this study, we used gene expression datasets from the GEO database to screen for potential biomarkers related to FOLFIRI treatment response in advanced colon cancer. Furthermore, we obtained clinical information and RNA sequencing data of colon cancer from TCGA, the GEPIA and the Human Atlas for verification.

We used the WGCNA to explore gene co-expression modules related to the response to FOLFIRI treatment in advanced colon cancer. Through the construction of the co-expression network, we finally screened out 19 modules. Based on the results of the correlation analysis, we screened out the blue modules with the highest correlation with chemotherapy response. We further screened 50 hub genes in the blue module and finally found the key genes AEBP1, BGN, and TAGLN that are negatively related to OS and DFS.

Adipocyte enhancer-binding protein 1 (AEBP1) acts as an essential inflammation regulator (Majdalawieh and Ro, 2010). Studies have shown that AEBP1 plays an important regulatory role in the pathogenesis of atherosclerosis and may be a potential marker for the prevention and treatment of atherosclerosis (Bogachev et al., 2011). It was reported that AEBP1 may participate in activating the NF-κB pathway by binding and inhibiting the activity of IK-BA, which further mediates the inflammatory response (Majdalawieh et al., 2020). In recent years, the role of AEBP1 in tumor regulation has gradually attracted people’s attention. In gliomas, AEBP1 was defined as a potential oncogenic driver, with potential implications for therapeutic intervention (Sinha et al., 2019). In addition, Holloway et al. found that AEBP1 was involved in the signaling pathway between epithelial cells and cell matrix, leading to the formation of the tumor inflammatory microenvironment (Holloway et al., 2012). In our study, we found that AEBP1 may be a predictive target for response after FOLFIRI treatment in colon cancer.

Biglycan (BGN) was found to be associated with tumor progression in gastric cancer (Hu et al., 2014), pancreatic adenocarcinoma (Aprile et al., 2013), endometrial cancer (Liu et al., 2014), and prostate cancer (Jacobsen et al., 2017). Moreover, studies have shown that high expression of BGN is associated with reduced overall survival in lung cancer (Morimoto et al., 2021). In colorectal cancer, Gu et al. found that high expression of BGN is associated with the tumor malignant phenotype (Gu et al., 2012). Our research suggests that BGN has an important predictive role in FOLFIRI response in advanced colon cancer. However, the mechanism of BGN in chemotherapy resistance of colon cancer has not been explored.

Transgelin (TAGLN) is a mesenchymal protein involved in the EMT process and plays a regulatory role in a variety of cancers (Lees-Miller et al., 1987; Thompson et al., 2012; Naito et al., 2014). It is worth noting that TAGLN seems to participate in promoting and suppressing cancer in regulating the progression of malignant tumors. For instance, TAGLN expression was found to be downregulated in breast ductal carcinoma (Wulfkuhle et al., 2002). Controversially, some studies found a positive correlation in colorectal cancer (Lin et al., 2009) and nerve sheath tumor (Park et al., 2014). The regulatory effect of TAGLN on colon cancer progression needs to be further explored. Recently, many studies have shown that chemotherapy combined with anti-tumor immunity can greatly improve the curative effect on tumors (Chasov et al., 2020). Free fluorouracil combined with anti-tumor immunity can significantly inhibit the growth of tumors in the mouse model of colon adenocarcinoma *in situ* (Guo et al., 2021). A randomized phase II clinical study showed that the combination of immune checkpoint inhibitors and cytotoxic drugs is an early effective strategy for the treatment of metastatic colon cancer (Antoniotti et al., 2020). A clinical retrospective study showed that even in patients with metastatic colon cancer with immune refractory microsatellite stability, the application of immune checkpoint inhibitors before chemotherapy can enhance the cytotoxicity (Martin-Romano et al., 2020). This shows that chemotherapy combined with immunotherapy can improve the chemotherapy effect on patients. The drug resistance core gene identified in this study may provide some guidance for the timing of chemotherapy combined with immunotherapy.

Our study has certain limitations. First of all, our results of the WGCNA can be biased or invalid when coping with technical artifacts or tissue contaminations. Second, in order to verify the credibility of the WGCNA results, we used the GEPIA database and the HPA database. Due to the limitations of the database, we cannot ensure that each tumor and normal sample were from the same patient. Third, as this study is based on the weighted gene co-expression network analysis of GSE62080, it is inevitable that some clinical covariates and potential confounding factors are not involved, which will cause certain bias to the research results.

In recent years, the co-expression analysis technology using large-scale datasets for multi-gene analysis is profound in the construction of cancer key gene expression networks in the research of malignant tumors. In this paper, we studied the correlation between genes, modules, and clinical characteristics through the gene co-expression network constructed by the WGCNA and successfully screened out three key genes (AEBP1, BGN, and TAGLN) related to the response after FOLFIRI. Our study provides potential predictive biological markers for the FOLFIRI response in advanced colon cancer.

## Data Availability

The datasets presented in this study can be found in online repositories. The names of the repository/repositories and accession number(s) can be found below: https://www.ncbi.nlm.nih.gov/ and GSE62080.
